# *r*-2,*c*-6-Bis(4-chloro­phen­yl)-*t*-3-isopropyl-1-nitro­sopiperidin-4-one

**DOI:** 10.1107/S1600536808029723

**Published:** 2008-09-20

**Authors:** P. Gayathri, A. Thiruvalluvar, A. Manimekalai, S. Sivakumar, R. J. Butcher

**Affiliations:** a PG Research Department of Physics, Rajah Serfoji Government College (Autonomous), Thanjavur 613 005, Tamilnadu, India; b Department of Chemistry, Annamalai University, Annamalai Nagar 608 002, Tamilnadu, India; c Department of Chemistry, Howard University, 525 College Street NW, Washington, DC 20059, USA

## Abstract

In the title mol­ecule, C_20_H_20_Cl_2_N_2_O_2_, the piperidine ring adopts a chair conformation and the nitroso group at position 1 has a bis­ectional orientation. The two benzene rings and the isopropyl group attached to the piperidine ring in positions 2, 6 and 3, respectively, have axial orientations. The dihedral angle between the two benzene rings is 21.56 (13)°. One of the Cl atoms is disordered over two positions in a 0.281 (5):0.719 (5) ratio. In the crystal structure, mol­ecules are linked by C—H⋯O hydrogen bonds and a short C—H⋯O contact occurs within the mol­ecule.

## Related literature

For related crystal structures, see: Balamurugan *et al.* (2006 ▶, 2007 ▶); Thiruvalluvar, Balamurugan, Jayabharathi & Manimekalai (2007 ▶); Thiruvalluvar, Balamurugan, Jayabharathi, Manimekalai & Rajarajan (2007 ▶).
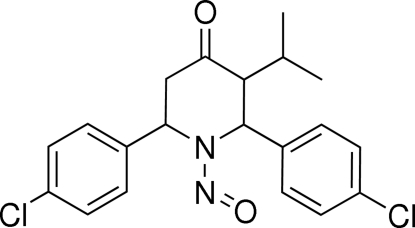


## Experimental

### 

#### Crystal data

C_20_H_20_Cl_2_N_2_O_2_
*M* *_r_* = 391.28
Triclinic, 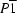

*a* = 8.2771 (2) Å
*b* = 11.1921 (4) Å
*c* = 11.2351 (4) Å
α = 93.375 (3)°
β = 106.924 (3)°
γ = 104.549 (3)°
*V* = 953.95 (6) Å^3^
*Z* = 2
Cu *K*α radiation
μ = 3.20 mm^−1^
*T* = 200 (2) K
0.54 × 0.47 × 0.41 mm

#### Data collection

Oxford Diffraction R Gemini diffractometer
Absorption correction: multi-scan (*CrysAlis RED*; Oxford Diffraction, 2008 ▶) *T*_min_ = 0.269, *T*_max_ = 1.000 (expected range = 0.073–0.270)
8014 measured reflections
3752 independent reflections
3506 reflections with *I* > 2σ(*I*)
*R*_int_ = 0.020

#### Refinement

*R*[*F* ^2^ > 2σ(*F* ^2^)] = 0.056
*wR*(*F* ^2^) = 0.155
*S* = 1.02
3752 reflections
239 parameters
2 restraints
H-atom parameters constrained
Δρ_max_ = 0.55 e Å^−3^
Δρ_min_ = −0.43 e Å^−3^

### 

Data collection: *CrysAlis CCD* (Oxford Diffraction, 2008 ▶); cell refinement: *CrysAlis RED* (Oxford Diffraction, 2008 ▶); data reduction: *CrysAlis RED*; program(s) used to solve structure: *SHELXS97* (Sheldrick, 2008 ▶); program(s) used to refine structure: *SHELXL97* (Sheldrick, 2008 ▶); molecular graphics: *ORTEP-3* (Farrugia, 1997 ▶); software used to prepare material for publication: *PLATON* (Spek, 2003 ▶).

## Supplementary Material

Crystal structure: contains datablocks global, I. DOI: 10.1107/S1600536808029723/hb2797sup1.cif

Structure factors: contains datablocks I. DOI: 10.1107/S1600536808029723/hb2797Isup2.hkl

Additional supplementary materials:  crystallographic information; 3D view; checkCIF report

## Figures and Tables

**Table 1:** Hydrogen-bond geometry (Å, °)

*D*—H⋯*A*	*D*—H	H⋯*A*	*D*⋯*A*	*D*—H⋯*A*
C2—H2⋯O11	1.00	2.24	2.676 (2)	105
C5—H5*B*⋯O4^i^	0.99	2.55	3.530 (3)	171
C32—H32*C*⋯O4^ii^	0.98	2.59	3.532 (3)	162

Symmetry codes: (i) 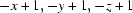; (ii) 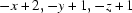.

**Fig. 1.**
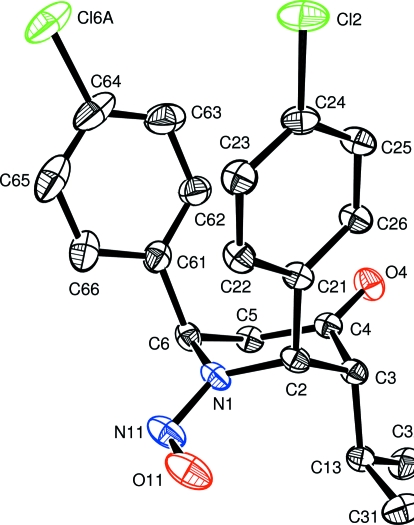
The molecular structure of (I) with displacement ellipsoids drawn at the 30% probability level. H atoms have been omitted for clarity.

**Fig. 2.**
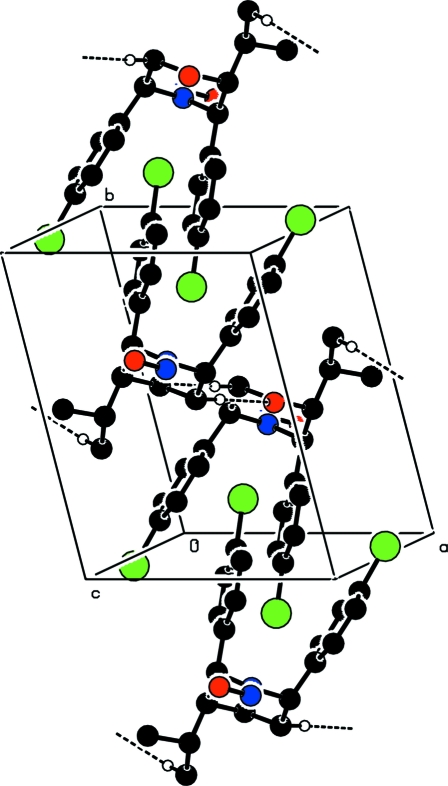
The packing of (I), viewed along the c axis. Dashed lines indicate hydrogen bonds. H atoms not involved in hydrogen bonding have been omitted.

## supplementary crystallographic information

### Comment

Various crystal structures of di-2-furylpiperidin-4-one derivatives have been reported, wherein the piperidine ring adopts a chair (Balamurugan *et al.*, 2006), a twist-boat (Balamurugan *et al.*, 2007) and a chair conformation (Thiruvalluvar, Balamurugan, Jayabharathi & Manimekalai, 2007). Thiruvalluvar, Balamurugan, Jayabharathi, Manimekalai & Rajarajan (2007) have reported the crystal structure of a diphenylpiperidin-4-ol derivative, wherein the piperidine ring adopts a chair conformation.

In the title molecule, C_20_H_20_Cl_2_N_2_O_2_ (Fig. 1), the piperidine ring adopts a chair conformation. The nitroso group at position 1 has a bisectional orientation. The two phenyl rings and the isopropyl group attached to the piperidine ring in positions 2, 6 and 3, respectively, have axial orientations. The dihedral angle between the two phenyl rings is 21.56 (13)°. Compound (I) is chiral: in the arbitrarily chosen asymmetric molecule, C2, C3 and C6 have S, R, and R conformations respectively, but crystal symmetry generates a racemic mixture. In the crystal, the molecules are linked by C—H···O hydrogen bonds (Fig. 2, Table 1).

### Experimental

To a solution of t3-isopropyl-r2,c6-bis(4-chlorophenyl) piperidin-4-one (1.81 g, 0.005 mol) in chloroform (10 ml), concentrated HCl (1.5 ml) and water (1.5 ml) were added. While stirring, solid NaNO_2_ (3 g, 0.012 mol) was added in small portions to the reaction mixture over a period of 30 minutes. The stirring was continued for another 30 minutes. The organic layer was washed with water and saturated NaHCO_3_ and dried over anhydrous Na_2_SO_4_. After the removal of chloroform, the crude solid was recrystallized from distilled ethanol, to yield 1.5 g of colourless chunks of (I) (yield = 76%).

### Refinement

The Cl atom attached to C64 is disordered over two positions in a 0.281 (5) to 0.719 (5) ratio. The ADPs of both chlorine atoms were set to be identical. The C—Cl distances were restrained to be 1.740 (3) Å. The H atoms were positioned geometrically (C–H = 0.95–1.00 Å) and refined as riding with *U*_iso_(H) = 1.2*U*_eq_(C) or 1.5U_eq_(methyl C).

### Crystal data

**Table:**

C_20_H_20_Cl_2_N_2_O_2_	*Z* = 2
*M**_r_* = 391.28	*F*(000) = 408
Triclinic, *P*1	*D*_x_ = 1.362 Mg m^−^^3^
Hall symbol: -P 1	Melting point: 371 K
*a* = 8.2771 (2) Å	Cu *K*α radiation, λ = 1.54184 Å
*b* = 11.1921 (4) Å	Cell parameters from 7346 reflections
*c* = 11.2351 (4) Å	θ = 4.1–73.2°
α = 93.375 (3)°	µ = 3.20 mm^−^^1^
β = 106.924 (3)°	*T* = 200 K
γ = 104.549 (3)°	Chunk, colourless
*V* = 953.95 (6) Å^3^	0.54 × 0.47 × 0.41 mm

### Data collection

**Table:**

Oxford Diffraction R Gemini diffractometer	3752 independent reflections
Radiation source: fine-focus sealed tube	3506 reflections with *I* > 2σ(*I*)
graphite	*R*_int_ = 0.020
Detector resolution: 10.5081 pixels mm^-1^	θ_max_ = 73.6°, θ_min_ = 4.1°
φ and ω scans	*h* = −9→10
Absorption correction: multi-scan (CrysAlis RED; Oxford Diffraction, 2008)	*k* = −13→13
*T*_min_ = 0.269, *T*_max_ = 1.000	*l* = −13→13
8014 measured reflections	

### Refinement

**Table:**

Refinement on *F*^2^	Primary atom site location: structure-invariant direct methods
Least-squares matrix: full	Secondary atom site location: difference Fourier map
*R*[*F*^2^ > 2σ(*F*^2^)] = 0.056	Hydrogen site location: inferred from neighbouring sites
*wR*(*F*^2^) = 0.155	H-atom parameters constrained
*S* = 1.02	*w* = 1/[σ^2^(*F*_o_^2^) + (0.0861*P*)^2^ + 0.6137*P*] where *P* = (*F*_o_^2^ + 2*F*_c_^2^)/3
3752 reflections	(Δ/σ)_max_ = 0.001
239 parameters	Δρ_max_ = 0.55 e Å^−^^3^
2 restraints	Δρ_min_ = −0.43 e Å^−^^3^

### Special details

**Table:**

Geometry. Bond distances, angles *etc*. have been calculated using the rounded fractional coordinates. All su's are estimated from the variances of the (full) variance-covariance matrix. The cell e.s.d.'s are taken into account in the estimation of distances, angles and torsion angles
Refinement. Refinement of *F*^2^ against ALL reflections. The weighted *R*-factor *wR* and goodness of fit *S* are based on *F*^2^, conventional *R*-factors *R* are based on *F*, with *F* set to zero for negative *F*^2^. The threshold expression of *F*^2^ > σ(*F*^2^) is used only for calculating *R*-factors(gt) *etc*. and is not relevant to the choice of reflections for refinement. *R*-factors based on *F*^2^ are statistically about twice as large as those based on *F*, and *R*- factors based on ALL data will be even larger.

### Fractional atomic coordinates and isotropic or equivalent isotropic displacement parameters (Å^2^)

**Table:**

	*x*	*y*	*z*	*U*_iso_*/*U*_eq_	Occ. (<1)
Cl2	0.43589 (10)	−0.19782 (5)	0.33972 (6)	0.0590 (2)	
Cl6A	−0.1316 (3)	−0.06761 (12)	0.2338 (3)	0.0841 (7)	0.719 (5)
O4	0.7087 (2)	0.46794 (17)	0.48136 (13)	0.0497 (5)	
O11	0.5501 (3)	0.32189 (19)	−0.05880 (16)	0.0660 (7)	
N1	0.4995 (2)	0.34969 (16)	0.11627 (14)	0.0372 (5)	
N11	0.4570 (3)	0.35585 (18)	−0.00614 (17)	0.0522 (6)	
C2	0.6545 (3)	0.31257 (18)	0.18850 (17)	0.0334 (5)	
C3	0.7723 (3)	0.42145 (18)	0.29227 (17)	0.0327 (5)	
C4	0.6642 (3)	0.45915 (17)	0.36822 (18)	0.0348 (6)	
C5	0.5004 (3)	0.49030 (18)	0.29305 (18)	0.0363 (6)	
C6	0.3862 (3)	0.39307 (19)	0.17854 (19)	0.0383 (6)	
C13	0.8596 (3)	0.53527 (19)	0.2373 (2)	0.0389 (6)	
C21	0.5990 (3)	0.18630 (18)	0.23147 (17)	0.0326 (5)	
C22	0.4852 (3)	0.08611 (19)	0.14198 (19)	0.0389 (6)	
C23	0.4339 (3)	−0.0315 (2)	0.1750 (2)	0.0421 (6)	
C24	0.4973 (3)	−0.04937 (19)	0.2980 (2)	0.0398 (6)	
C25	0.6100 (3)	0.0473 (2)	0.38825 (19)	0.0409 (6)	
C26	0.6609 (3)	0.16493 (19)	0.35455 (18)	0.0384 (6)	
C31	0.9944 (4)	0.5055 (3)	0.1809 (3)	0.0592 (9)	
C32	0.9513 (3)	0.6478 (2)	0.3399 (3)	0.0543 (8)	
C61	0.2563 (3)	0.2832 (2)	0.2041 (2)	0.0421 (6)	
C62	0.2692 (3)	0.2498 (2)	0.3220 (3)	0.0524 (8)	
C63	0.1510 (4)	0.1436 (3)	0.3368 (4)	0.0703 (10)	
C64	0.0189 (3)	0.0718 (2)	0.2332 (4)	0.0720 (12)	
C65	0.0003 (3)	0.1074 (3)	0.1156 (4)	0.0746 (12)	
C66	0.1184 (3)	0.2115 (3)	0.1008 (3)	0.0593 (9)	
Cl6B	−0.0853 (7)	−0.0526 (4)	0.2990 (7)	0.0841 (7)	0.281 (5)
H2	0.72236	0.30238	0.12995	0.0401*	
H3	0.86751	0.39229	0.34954	0.0393*	
H5A	0.53537	0.57198	0.26429	0.0435*	
H5B	0.42922	0.49868	0.34856	0.0435*	
H6	0.31545	0.43675	0.11767	0.0459*	
H13	0.76630	0.55652	0.16987	0.0467*	
H22	0.44203	0.09866	0.05694	0.0467*	
H23	0.35584	−0.09911	0.11325	0.0505*	
H25	0.65284	0.03391	0.47303	0.0491*	
H26	0.73940	0.23186	0.41688	0.0461*	
H31A	0.93640	0.43346	0.11470	0.0887*	
H31B	1.08772	0.48622	0.24672	0.0887*	
H31C	1.04551	0.57768	0.14499	0.0887*	
H32A	0.86543	0.66784	0.37622	0.0814*	
H32B	1.00279	0.71949	0.30348	0.0814*	
H32C	1.04446	0.62838	0.40568	0.0814*	
H62	0.35940	0.29957	0.39345	0.0629*	
H63	0.16109	0.12048	0.41808	0.0844*	
H65	−0.09399	0.06000	0.04494	0.0896*	
H66	0.10616	0.23497	0.01945	0.0712*	

### Atomic displacement parameters (Å^2^)

**Table:**

	*U*^11^	*U*^22^	*U*^33^	*U*^12^	*U*^13^	*U*^23^
Cl2	0.0920 (5)	0.0271 (3)	0.0621 (4)	0.0080 (3)	0.0375 (3)	0.0087 (2)
Cl6A	0.0520 (8)	0.0412 (5)	0.157 (2)	−0.0006 (5)	0.0433 (11)	0.0067 (8)
O4	0.0592 (10)	0.0591 (10)	0.0295 (7)	0.0196 (8)	0.0111 (7)	−0.0008 (6)
O11	0.1101 (16)	0.0592 (11)	0.0380 (9)	0.0351 (11)	0.0273 (10)	0.0098 (8)
N1	0.0515 (10)	0.0316 (8)	0.0251 (7)	0.0123 (7)	0.0063 (7)	0.0056 (6)
N11	0.0833 (14)	0.0385 (10)	0.0322 (9)	0.0179 (10)	0.0137 (9)	0.0057 (7)
C2	0.0445 (10)	0.0299 (9)	0.0274 (9)	0.0122 (8)	0.0124 (8)	0.0045 (7)
C3	0.0389 (10)	0.0293 (9)	0.0299 (9)	0.0105 (8)	0.0096 (7)	0.0058 (7)
C4	0.0428 (10)	0.0264 (9)	0.0327 (10)	0.0067 (8)	0.0114 (8)	0.0017 (7)
C5	0.0441 (11)	0.0289 (9)	0.0366 (10)	0.0110 (8)	0.0137 (8)	0.0024 (8)
C6	0.0430 (11)	0.0336 (10)	0.0354 (10)	0.0143 (8)	0.0050 (8)	0.0065 (8)
C13	0.0398 (10)	0.0343 (10)	0.0422 (10)	0.0093 (8)	0.0119 (8)	0.0132 (8)
C21	0.0407 (10)	0.0282 (9)	0.0322 (9)	0.0130 (8)	0.0139 (8)	0.0038 (7)
C22	0.0481 (11)	0.0347 (10)	0.0328 (10)	0.0133 (9)	0.0101 (8)	0.0022 (8)
C23	0.0489 (12)	0.0308 (10)	0.0425 (11)	0.0083 (9)	0.0125 (9)	−0.0034 (8)
C24	0.0526 (12)	0.0266 (9)	0.0471 (11)	0.0118 (8)	0.0251 (9)	0.0070 (8)
C25	0.0570 (12)	0.0333 (10)	0.0346 (10)	0.0134 (9)	0.0164 (9)	0.0083 (8)
C26	0.0503 (11)	0.0295 (10)	0.0329 (10)	0.0093 (8)	0.0114 (8)	0.0026 (7)
C31	0.0607 (15)	0.0555 (15)	0.0714 (17)	0.0117 (12)	0.0389 (13)	0.0145 (12)
C32	0.0528 (13)	0.0332 (11)	0.0700 (16)	0.0032 (10)	0.0170 (12)	0.0066 (10)
C61	0.0360 (10)	0.0342 (10)	0.0547 (12)	0.0132 (8)	0.0095 (9)	0.0065 (9)
C62	0.0463 (12)	0.0490 (13)	0.0657 (15)	0.0140 (10)	0.0207 (11)	0.0186 (11)
C63	0.0632 (17)	0.0625 (17)	0.107 (2)	0.0285 (14)	0.0448 (17)	0.0408 (17)
C64	0.0418 (13)	0.0347 (13)	0.148 (3)	0.0124 (10)	0.0409 (17)	0.0148 (16)
C65	0.0403 (13)	0.0477 (15)	0.120 (3)	0.0057 (11)	0.0118 (15)	−0.0104 (16)
C66	0.0446 (13)	0.0496 (14)	0.0720 (17)	0.0135 (11)	0.0026 (11)	−0.0012 (12)
Cl6B	0.0520 (8)	0.0412 (5)	0.157 (2)	−0.0006 (5)	0.0433 (11)	0.0067 (8)

### Geometric parameters (Å, °)

**Table:**

Cl2—C24	1.745 (2)	C62—C63	1.390 (4)
Cl6A—C64	1.737 (3)	C63—C64	1.380 (5)
Cl6B—C64	1.766 (6)	C64—C65	1.379 (6)
O4—C4	1.207 (2)	C65—C66	1.373 (5)
O11—N11	1.214 (3)	C2—H2	1.0000
N1—N11	1.327 (2)	C3—H3	1.0000
N1—C2	1.476 (3)	C5—H5A	0.9900
N1—C6	1.475 (3)	C5—H5B	0.9900
C2—C21	1.525 (3)	C6—H6	1.0000
C2—C3	1.539 (3)	C13—H13	1.0000
C3—C4	1.516 (3)	C22—H22	0.9500
C3—C13	1.555 (3)	C23—H23	0.9500
C4—C5	1.510 (3)	C25—H25	0.9500
C5—C6	1.531 (3)	C26—H26	0.9500
C6—C61	1.519 (3)	C31—H31A	0.9800
C13—C32	1.527 (3)	C31—H31B	0.9800
C13—C31	1.527 (4)	C31—H31C	0.9800
C21—C22	1.393 (3)	C32—H32A	0.9800
C21—C26	1.386 (3)	C32—H32B	0.9800
C22—C23	1.385 (3)	C32—H32C	0.9800
C23—C24	1.374 (3)	C62—H62	0.9500
C24—C25	1.372 (3)	C63—H63	0.9500
C25—C26	1.388 (3)	C65—H65	0.9500
C61—C66	1.397 (4)	C66—H66	0.9500
C61—C62	1.379 (4)		
			
N11—N1—C2	123.99 (18)	C21—C2—H2	107.00
N11—N1—C6	114.35 (18)	C2—C3—H3	108.00
C2—N1—C6	121.50 (15)	C4—C3—H3	108.00
O11—N11—N1	115.3 (2)	C13—C3—H3	108.00
N1—C2—C3	108.06 (17)	C4—C5—H5A	109.00
N1—C2—C21	110.95 (19)	C4—C5—H5B	109.00
C3—C2—C21	116.29 (15)	C6—C5—H5A	109.00
C2—C3—C4	109.5 (2)	C6—C5—H5B	109.00
C2—C3—C13	111.85 (16)	H5A—C5—H5B	108.00
C4—C3—C13	110.24 (17)	N1—C6—H6	107.00
O4—C4—C3	122.5 (2)	C5—C6—H6	107.00
O4—C4—C5	122.1 (2)	C61—C6—H6	107.00
C3—C4—C5	115.41 (17)	C3—C13—H13	109.00
C4—C5—C6	113.82 (18)	C31—C13—H13	109.00
N1—C6—C5	109.7 (2)	C32—C13—H13	109.00
N1—C6—C61	110.74 (17)	C21—C22—H22	119.00
C5—C6—C61	115.49 (18)	C23—C22—H22	119.00
C3—C13—C31	110.9 (2)	C22—C23—H23	120.00
C3—C13—C32	110.29 (18)	C24—C23—H23	120.00
C31—C13—C32	109.0 (2)	C24—C25—H25	120.00
C2—C21—C22	118.53 (17)	C26—C25—H25	120.00
C2—C21—C26	123.28 (18)	C21—C26—H26	119.00
C22—C21—C26	118.15 (18)	C25—C26—H26	119.00
C21—C22—C23	121.07 (19)	C13—C31—H31A	109.00
C22—C23—C24	119.3 (2)	C13—C31—H31B	109.00
Cl2—C24—C23	119.49 (17)	C13—C31—H31C	109.00
Cl2—C24—C25	119.45 (16)	H31A—C31—H31B	109.00
C23—C24—C25	121.1 (2)	H31A—C31—H31C	109.00
C24—C25—C26	119.39 (19)	H31B—C31—H31C	109.00
C21—C26—C25	121.06 (19)	C13—C32—H32A	109.00
C6—C61—C62	123.8 (2)	C13—C32—H32B	109.00
C6—C61—C66	117.3 (2)	C13—C32—H32C	109.00
C62—C61—C66	118.9 (2)	H32A—C32—H32B	109.00
C61—C62—C63	120.4 (3)	H32A—C32—H32C	110.00
C62—C63—C64	119.9 (3)	H32B—C32—H32C	109.00
Cl6A—C64—C63	125.8 (3)	C61—C62—H62	120.00
Cl6A—C64—C65	114.0 (3)	C63—C62—H62	120.00
C63—C64—C65	120.2 (3)	C62—C63—H63	120.00
Cl6B—C64—C63	102.5 (4)	C64—C63—H63	120.00
Cl6B—C64—C65	137.3 (4)	C64—C65—H65	120.00
C64—C65—C66	119.9 (3)	C66—C65—H65	120.00
C61—C66—C65	120.7 (3)	C61—C66—H66	120.00
N1—C2—H2	107.00	C65—C66—H66	120.00
C3—C2—H2	107.00		
			
C2—N1—N11—O11	3.9 (3)	C3—C4—C5—C6	48.5 (2)
C6—N1—N11—O11	179.18 (19)	C4—C5—C6—N1	−40.6 (2)
N11—N1—C2—C3	120.4 (2)	C4—C5—C6—C61	85.3 (3)
N11—N1—C2—C21	−111.1 (2)	N1—C6—C61—C62	107.0 (3)
C6—N1—C2—C3	−54.6 (2)	N1—C6—C61—C66	−71.9 (3)
C6—N1—C2—C21	73.9 (2)	C5—C6—C61—C62	−18.5 (3)
N11—N1—C6—C5	−127.96 (18)	C5—C6—C61—C66	162.8 (2)
N11—N1—C6—C61	103.4 (2)	C2—C21—C22—C23	178.3 (2)
C2—N1—C6—C5	47.5 (2)	C26—C21—C22—C23	0.5 (4)
C2—N1—C6—C61	−81.1 (2)	C2—C21—C26—C25	−178.2 (2)
N1—C2—C3—C4	53.3 (2)	C22—C21—C26—C25	−0.5 (4)
N1—C2—C3—C13	−69.2 (2)	C21—C22—C23—C24	−0.3 (4)
C21—C2—C3—C4	−72.2 (2)	C22—C23—C24—Cl2	−179.0 (2)
C21—C2—C3—C13	165.3 (2)	C22—C23—C24—C25	0.1 (4)
N1—C2—C21—C22	52.8 (3)	Cl2—C24—C25—C26	179.0 (2)
N1—C2—C21—C26	−129.5 (2)	C23—C24—C25—C26	−0.1 (4)
C3—C2—C21—C22	176.8 (2)	C24—C25—C26—C21	0.3 (4)
C3—C2—C21—C26	−5.5 (4)	C6—C61—C62—C63	−176.2 (3)
C2—C3—C4—O4	127.9 (2)	C66—C61—C62—C63	2.6 (4)
C2—C3—C4—C5	−54.5 (2)	C6—C61—C66—C65	177.0 (3)
C13—C3—C4—O4	−108.6 (2)	C62—C61—C66—C65	−1.8 (4)
C13—C3—C4—C5	68.9 (2)	C61—C62—C63—C64	−0.7 (5)
C2—C3—C13—C31	−68.1 (3)	C62—C63—C64—Cl6A	176.3 (3)
C2—C3—C13—C32	171.1 (2)	C62—C63—C64—C65	−2.2 (5)
C4—C3—C13—C31	169.8 (2)	Cl6A—C64—C65—C66	−175.6 (3)
C4—C3—C13—C32	49.0 (3)	C63—C64—C65—C66	3.0 (5)
O4—C4—C5—C6	−134.0 (2)	C64—C65—C66—C61	−1.0 (4)

### Hydrogen-bond geometry (Å, °)

**Table:**

*D*—H···*A*	*D*—H	H···*A*	*D*···*A*	*D*—H···*A*
C2—H2···O11	1.00	2.24	2.676 (2)	105
C5—H5B···O4^i^	0.99	2.55	3.530 (3)	171
C32—H32C···O4^ii^	0.98	2.59	3.532 (3)	162

Symmetry codes: (i) −*x*+1, −*y*+1, −*z*+1; (ii) −*x*+2, −*y*+1, −*z*+1.
